# An updated nomenclature for plant ribosomal protein genes

**DOI:** 10.1093/plcell/koac333

**Published:** 2022-11-25

**Authors:** M Regina Scarpin, Michael Busche, Ryan E Martinez, Lisa C Harper, Leonore Reiser, Dóra Szakonyi, Catharina Merchante, Ting Lan, Wei Xiong, Beixin Mo, Guiliang Tang, Xuemei Chen, Julia Bailey-Serres, Karen S Browning, Jacob O Brunkard

**Affiliations:** Laboratory of Genetics, University of Wisconsin – Madison, Madison, Wisconsin 53706, USA; Department of Plant and Microbial Biology, University of California – Berkeley, Berkeley, California 94720, USA; Plant Gene Expression Center, USDA Agricultural Research Service, Albany, California 94710, USA; Laboratory of Genetics, University of Wisconsin – Madison, Madison, Wisconsin 53706, USA; Laboratory of Genetics, University of Wisconsin – Madison, Madison, Wisconsin 53706, USA; Corn Insects and Crop Genetics Research Unit, USDA Agricultural Research Service, Ames, Iowa 50011, USA; The Arabidopsis Information Resource, Phoenix Bioinformatics, Fremont, California 94538, USA; Plant Molecular Biology, Instituto Gulbenkian de Ciência, 2780-156 Oeiras, Portugal; Departamento de Biología Molecular y Bioquímica, Instituto de Hortofruticultura Subtropical y Mediterránea “La Mayora” (IHSM-UMA-CSIC), Facultad de Ciencias, Campus, de Teatinos, Universidad de Málaga, 29071 Málaga, Spain; Guangdong Provincial Key Laboratory for Plant Epigenetics, Longhua Bioindustry and Innovation Research Institute, College of Life Sciences and Oceanography, Shenzhen University, Shenzhen 518060, China; Key Laboratory of Optoelectronic Devices and Systems of Ministry of Education and Guangdong Province, College of Optoelectronic Engineering, Shenzhen University, Shenzhen 518060, China; Guangdong Provincial Key Laboratory for Plant Epigenetics, Longhua Bioindustry and Innovation Research Institute, College of Life Sciences and Oceanography, Shenzhen University, Shenzhen 518060, China; Guangdong Provincial Key Laboratory for Plant Epigenetics, Longhua Bioindustry and Innovation Research Institute, College of Life Sciences and Oceanography, Shenzhen University, Shenzhen 518060, China; Department of Biological Sciences, Life Science and Technology Institute, Michigan Technological University, Houghton, Michigan 49931, USA; Department of Botany and Plant Sciences and Center for Plant Cell Biology, Institute of Integrative Genome Biology, University of California – Riverside, Riverside, California 92521, USA; Department of Botany and Plant Sciences and Center for Plant Cell Biology, Institute of Integrative Genome Biology, University of California – Riverside, Riverside, California 92521, USA; Department of Molecular Biosciences, University of Texas, Austin, Texas 78712, USA; Laboratory of Genetics, University of Wisconsin – Madison, Madison, Wisconsin 53706, USA; Department of Plant and Microbial Biology, University of California – Berkeley, Berkeley, California 94720, USA; Plant Gene Expression Center, USDA Agricultural Research Service, Albany, California 94710, USA

Dear Editor,

Across all living organisms, ribosomes are large macromolecular complexes that synthesize proteins by translating messenger RNA codes into amino acid sequences. Structurally, ribosomes are composed of ∼50–80 ribosomal proteins (r-proteins) and 3 or 4 ribosomal RNAs (rRNAs). Over the past 4 billion years, ribosomes have evolved some differences in rRNA and r-protein composition, with certain subunits specific to bacteria, archaea and eukaryotes, plastids, or mitochondria, although many subunits are universally conserved with clear homology across all of life. Historically, the nomenclature of r-proteins was different in each species investigated, based on certain biochemical properties; that is, they were numbered in the order that they were separated by electrophoresis and/or chromatography (e.g., see Wittmann et al., 1971), rather than named for structural homology or function. The different naming systems fostered confusion for researchers, especially scientists not directly investigating ribosome biology, and hindered computational efforts to collate information on homologous r-proteins.

Ban et al. (2014) proposed to rectify these issues with a nomenclature for ribosomal proteins (r-proteins) that reflects the current understanding of ribosomal protein evolution. In the past few years, this nomenclature has been widely adopted among biomedical researchers and microbiologists. This homology-based r-protein nomenclature has not been as widely adopted among plant biologists, however, presumably because r-protein nomenclature is much more complicated in plants due to gene duplication. Here, we propose compatible upgrades to the homology-guided nomenclature proposed by Ban et al. (2014) so that this naming system can be adopted for widespread use in the plant biology community. We note that Lan et al. (2022) recently proposed updated nomenclature for plant cytosolic ribosomal proteins, focused on Arabidopsis and rice. The nomenclature outlined here is an extension of that proposed by Lan et al. (2022), expanding to include organellar ribosomes and additional species, with the intent that this nomenclature can serve as a template to guide future plant genome annotations. A more detailed comparison highlighting how this naming system builds on the Ban et al. (2014) and Lan et al. (2022) nomenclatures is offered below. Moreover, although we intend that this nomenclature can be universally adopted by plant biologists and curators, we also recognize that databases should maintain complete lists of alternative aliases for genes based on past nomenclatures, and we encourage authors to at least parenthetically mention past gene symbol aliases in their manuscripts. Alongside the new gene symbols, we urge authors and editors to clearly list the stable unique gene ID assigned by community databases and associated genome version numbers, such as the Arabidopsis Genome Initiative (AGI) locus code available at The Arabidopsis Information Resource (TAIR) and genome version (e.g., TAIR10).

In most lineages other than plants, r-proteins are encoded by single-copy genes (Steel and Jacobson, 1986; Uechi et al., 2001). There are some small exceptions, of course; for example, bacterial genomes often include a couple of duplicated r-protein genes (Yutin et al., 2012), including *E. coli*, which has two copies of *bL31* and two copies of *bL36* (Makarova et al., 2001). *S. cerevisiae*, a descendent of a recent whole-genome duplication event, has two homoeologous copies of many r-protein genes (Mager et al., 1997). Plant genomes, in contrast, almost always encode multiple paralogous copies of r-protein genes. For example, in *Arabidopsis thaliana*, every cytosolic r-protein is encoded by at least two paralogs, and several are encoded by five or six paralogs (Barakat et al., 2001; Salih et al., 2020; Lan et al., 2022). Moreover, plants also encode an additional two sets of r-proteins that localize in mitochondria or plastids to translate the organellar genomes. In sum, the Arabidopsis genome includes nearly 400 genes that encode r-proteins, about four times more than the ∼100 genes that encode r-proteins in mammals.

In consultation with The Arabidopsis Information Resource (TAIR), Maize Genetics and Genomics Database (MaizeGDB), and colleagues in the plant ribosome biology field, we propose new names and symbols for all of the r-proteins encoded by the Arabidopsis, tomato, maize, and rice genomes, which we intend will serve as a template to guide future plant genome annotations (Figure 1; Supplemental Data Set S1). We expect that this new nomenclature will enable greater communication with the wider audience of molecular biologists studying ribosomes and translation beyond plant biology.

**Figure 1:**
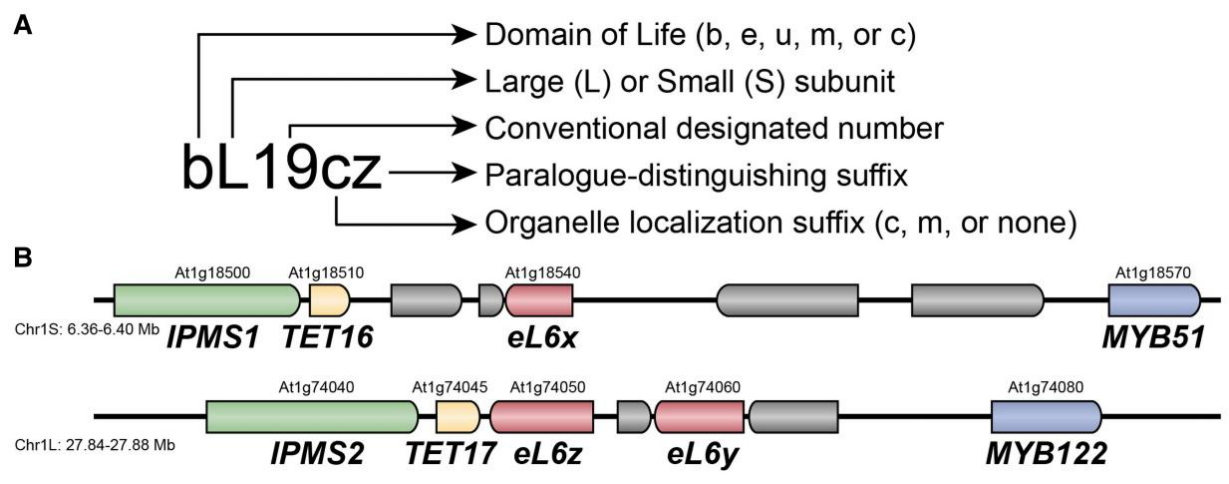
The proposed r-protein nomenclature follows standard rules across all domains of life to indicate homology of ribosomal subunits. A, The first letter indicates whether the r-protein is specific to bacterial genomes (b), archaean/eukaryotic genomes (e), or universal across genomes (u). In cases when the organellar r-protein has no cytosolic r-protein orthologues, the first letter instead indicates that the r-protein is specific to mitochondria (m) or plastids (c). The second letter indicates whether the r-protein is associated with the large 60S (L) or small 40S (S) subunit. The subunit number is based on consensus convention across model species as previously established (Ban et al., 2014). r-proteins that localize to plastids (c) or mitochondria (m) are indicated with a suffix, and this suffix is uppercase when the r-protein is encoded by the organellar genome. The final suffix is used to distinguish paralogs that encode homologous r-proteins within a genome. B, Representative example of r-protein paralogy in the *Arabidopsis thaliana* genome. *eL6x* is a homoeolog of two tandemly duplicated paralogs, *eL6z* and *eL6y*. Neighboring homoeologous genes and chromosomal locations are indicated to demonstrate synteny among these r-protein genes.

The r-protein nomenclature established by Ban et al. (2014) begins with a lowercase letter indicating whether the r-protein is specific to bacteria (with the letter “b”), archaea and eukaryotes (with the letter “e”), or all domains of life (with the letter “u” for “universal”). This is followed by either L or S to indicate whether the protein is a subunit of the large or small ribosomal subunit, respectively, and then by a number to specify the r-protein identity (Figure 1A). Cytosolic r-proteins have no suffix, whereas organelle-targeted r-protein symbols conclude with a suffix to indicate that they are targeted to mitochondria (with the letter “m”) or plastids (with the letter “c”, for “chloroplast”) (Bieri et al., 2017; Waltz et al., 2020, 2021). Organellar ribosomes have evolved unique r-protein subunits with no homology to cytosolic r-proteins; in these cases, the lowercase prefix indicates that the r-protein is targeted to mitochondria (with the letter “m”) or plastids (with the letter “c”, for “chloroplast”), and no suffix is added to show their subcellular localization (Bieri et al., 2017; Waltz et al., 2019, 2020, 2021).

Where feasible, the new r-protein symbols retain their traditional numbers—for example, archaeal/eukaryotic RPS6 is now eS6. Bacterial RPS6 is not homologous to eukaryotic RPS6, however, which previously caused some confusion; now, bacterial RPS6 is bS6, to indicate that it is not related to any archaeal/eukaryotic r-protein. Conversely, uS8 is now the universal symbol for bacterial r-protein S8, yeast r-protein S22, and human r-protein S15A, which all had different names despite their homology. Plant r-proteins occasionally have their own names, as well; for example, uL3, which was previously called L3 in bacteria, humans, and yeast, is called RIBOSOMAL PROTEIN1 (RP1) in Arabidopsis. Many Arabidopsis cytosolic r-proteins were first characterized from genetic screens for developmental defects, and the genes encoding these proteins were first named according to their mutant phenotypes, such as *apiculata*, *embryo defective*, *evershed*, *hapless*, *oligocellula*, *piggyback*, *pointed first leaves*, *short valve*, and *suppressor of acaulis*. Bifunctional r-proteins, such as eL40, which is proteolytically cleaved during ribosome assembly to separate the mature eL40 protein and its fused ubiquitin domain, are occasionally named not for the r-protein subunit, but for ubiquitin (in Arabidopsis, eL40 is called UBIQUITIN EXTENSION PROTEIN or UBQ, for example). These examples clearly illustrate the need for the new, unifying nomenclature for r-proteins in plant genomes so that our community can engage with other biologists.

Nonetheless, for continuity, past r-protein names and symbols should be maintained in databases as aliases. Moreover, we recommend that aliases should also be mentioned parenthetically as alternative gene names and symbols in future publications to ensure clarity for readers, e.g., “We detected that phosphorylation of r-protein eS6z (RPS6a) was reduced by rapamycin…”. This way, readers more familiar with the acronym “RP” to indicate “ribosomal protein” will not be confused by the new names, but the updated nomenclature will reconcile with the established nomenclature in other fields.

Animal r-proteins are encoded exclusively by the nuclear genome, so biomedical researchers have not emphasized the genomic location of r-protein genes in recent nomenclatures. Plant r-proteins, however, can be encoded by the nuclear, mitochondrial, or plastid genomes, with some variation in the location of these genes across species. There is even a special case, mitochondrial uL2, which has split into two genes in plants: the nucleus encodes a polypeptide homologous to the C-terminus of uL2 and the plastid encodes a polypeptide homologous to the N-terminal portion of uL2. To indicate cases when an r-protein is encoded by the organellar genome, we recommend using uppercase letters for the suffix (i.e., “M” and “C”) in publications.

The greatest challenge in adopting this new nomenclature for plant biology is how to best indicate paralogy of r-proteins (Figure 1B). In the simplest cases, there are only two paralogs, which could be designated with a single letter in alphabetical order, e.g., eS6a and eS6b. But in many cases, there are at least three paralogs, which is problematic because the plastid-targeted proteins are designated with a “c” (Bieri et al., 2017). In Arabidopsis, about 20 cytosolic r-proteins would end with a “c” and thus would be confused with the homologous plastid-targeted r-proteins that would also end with a “c”. There are many possible solutions to this problem, including several proposals advanced by members of the plant biology community; the most straightforward options are (1) to switch from a “c” designating chloroplast-targeted to a “p” designating plastid-targeted, (2) to add a hyphen separating the paralog designation from the protein symbol, (3) to distinguish between majuscule (uppercase) and miniscule (lowercase) lettering, such that “C” indicates a third paralog but “c” indicates plastid localization, (4) to use an alternative alphabet, such as Greek letters, to indicate paralogs, (5) to move the organelle indicator before the r-protein symbol, or (6) to start from the end of the alphabet, naming paralogs, e.g., uL15z, uL15y, uL15x.

After soliciting community feedback through a preprint version of this letter, social media, e-mails to additional community members, and the Plant Biology 2022 conference, we came to prefer the last option for several reasons. First, there is already literature on chloroplast ribosomes using the “c” to indicate plastid-targeted r-proteins, and there is considerable literature placing “m” or “c” at the end of the r-protein symbol to indicate organelle-targeting, so changing these would not serve the larger purpose of reaching a consensus nomenclature with r-protein biologists in other fields. Second, “p” is used as a suffix in many nomenclatures to distinguish proteins from nucleic acids (e.g., Tor1p is the protein encoded by the gene *tor1* in fission yeast) or to designate protein phosphorylation (e.g., rpS6P is phosphorylated eS6). Third, hyphens are typically used in plant nomenclatures to indicate alleles, so naming genes *eS6-a* and *eS6-b* could give the false impression that these are two alleles of a single gene, rather than paralogs. Fourth, relying on uppercase versus lowercase letters or on non-standard alphabets would require that database curators, computational biologists annotating new genomes, journal editors, and ribosome biologists working outside plant biology all pay strict attention to a slight typographical difference or expand the standard alphabet to accommodate this one set of genes, whereas starting from the end of the alphabet avoids any potential confusion.

We have provided a provisional table of r-protein names and symbols for Arabidopsis, tomato, maize, and rice for the plant biology community to consider, alongside their historical symbols in Arabidopsis and their symbols as recently proposed by Lan et al. (2022) (Supplemental Dataset S1). Note that the Lan et al. (2022) nomenclature differs primarily in how paralogs are indicated, which is a result of the exclusive focus of that nomenclature on cytosolic ribosomes. The new nomenclature will be added to public databases, including TAIR, MaizeGDB, and the Plant Cytoplasmic Ribosomal Proteins database (PlantCRP.cn). Previous names and symbols will be retained at these databases as a reference, and, as stated above, in publications, systematic identifiers (e.g., the AGI locus ID) should always be used alongside the updated r-protein symbols. We strongly encourage researchers to adopt the revised nomenclature to facilitate communication with researchers outside the plant community and increase the impact of our community's work on ribosome biology.

## Supplemental data

The following materials are available in the online version of this article.

Supplemental Dataset S1. The updated ribosomal protein nomenclature for select model species.

## Supplementary Material

koac333_Supplementary_Data

## References

[koac333-B100] Ban N , Beckmann R, Cate JHD, Dinman JD, Dragon F, Ellis SR, Lafontaine DLJ, Lindahl L **, Liljas A, Lipton JM,** et al. (2014) A new system for naming ribosomal proteins. Curr Opin Struct Biol 24: 165–169 24524803 10.1016/j.sbi.2014.01.002 PMC4358319

[koac333-B1] Barakat A , Szick-Miranda K, Chang IF, Guyot R, Blanc G, Cooke R, Delseny M, Bailey-Serres J (2001) The organization of cytoplasmic ribosomal protein genes in the Arabidopsis genome. Plant Physiol 127(2): 398–415 11598216 PMC125077

[koac333-B2] Bieri P , Leibundgut M, Saurer M, Boehringer D, Ban N (2017) The complete structure of the chloroplast 70S ribosome in complex with translation factor pY. EMBO J 36(4): 475–486 28007896 10.15252/embj.201695959 PMC5694952

[koac333-B3] Lan T , Xiong W, Chen X, Mo B, Tang G (2022) Plant cytoplasmic ribosomal proteins: an update on classification, nomenclature, evolution and resources. Plant J 110(1):292–318 35000252 10.1111/tpj.15667

[koac333-B4] Mager WH , Planta RJ, Ballesta JPG, Lee JC, Mizuta K, Suzuki K, Warner JR, Woolford J (1997) A new nomenclature for the cytoplasmic ribosomal proteins of *Saccharomyces cerevisiae*. Nucleic Acids Res 25(24): 4872–4875 9396790 10.1093/nar/25.24.4872 PMC147144

[koac333-B5] Makarova KS , Ponomarev VA, Koonin EV (2001) Two C or not two C: recurrent disruption of Zn-ribbons, gene duplication, lineage-specific gene loss, and horizontal gene transfer in evolution of bacterial ribosomal proteins. Genome Biol 2(9):RESEARCH 0033 11574053 10.1186/gb-2001-2-9-research0033 PMC56895

[koac333-B6] Salih KJ , Duncan O, Li L, Trösch J, Millar AH (2020) The composition and turnover of the *Arabidopsis thaliana* 80S cytosolic ribosome. Biochem J 477(16): 3019–3032 32744327 10.1042/BCJ20200385 PMC7452503

[koac333-B7] Steel LF , Jacobson A (1986) Ribosomal proteins are encoded by single copy genes in *Dictyostelium discoideum*. Gene 41(2–3): 165–172 3011594 10.1016/0378-1119(86)90095-8

[koac333-B8] Uechi T , Tanaka T, Kenmochi N (2001) A complete map of the human ribosomal protein genes: assignment of 80 genes to the cytogenetic map and implications for human disorders. Genomics 72(3): 223–230 11401437 10.1006/geno.2000.6470

[koac333-B9] Waltz F , Nguyen T-T, Arrivé M, Bochler A, Chicher J, Hammann P, Kuhn L, Quadrado M, Mireau H, Hashem Y, et al (2019) Small is big in Arabidopsis mitochondrial ribosome. Nat Plants 5(1): 106–117 30626926 10.1038/s41477-018-0339-y

[koac333-B10] Waltz F , Salinas-Giegé T, Englmeier R, Meichel H, Soufari H, Kuhn L, Pfeffer S, Förster F, Engel BD, Giegé P, et al (2021) How to build a ribosome from RNA fragments in Chlamydomonas mitochondria. Nat Commun 12(1): 7176 34887394 10.1038/s41467-021-27200-z PMC8660880

[koac333-B11] Waltz F , Soufari H, Bochler A, Giegé P, Hashem Y (2020) Cryo-EM structure of the RNA-rich plant mitochondrial ribosome. Nat Plants 6(4): 377–383 32251374 10.1038/s41477-020-0631-5

[koac333-B12] Wittmann HG , Stöffler G, Hindennach I, Kurland CG, Randall-Hazelbauer L, Birge EA, Nomura M, Kaltschmidt E, Mizushima S, Traut RR, et al (1971) Correlation of 30S ribosomal proteins of *Escherichia coli* isolated in different laboratories. MGG Mol Gen Genet 111(4): 327–333 4998702 10.1007/BF00569784

[koac333-B13] Yutin N , Puigbò P, Koonin E V, Wolf YI (2012) Phylogenomics of prokaryotic ribosomal proteins. PLoS ONE 7(5):e36972 22615861 10.1371/journal.pone.0036972 PMC3353972

